# Microbial Inactivation: Gaseous or Aqueous Ozonation?

**DOI:** 10.1021/acs.iecr.2c01551

**Published:** 2022-07-01

**Authors:** Emmanuel I. Epelle, Amy Emmerson, Marija Nekrasova, Andrew Macfarlane, Michael Cusack, Anthony Burns, William Mackay, Mohammed Yaseen

**Affiliations:** † School of Computing, Engineering & Physical Sciences, University of the West of Scotland, Paisley PA1 2BE, U.K.; ‡ School of Health & Life Sciences, University of the West of Scotland, Paisley PA1 2BE, U.K.; § ACS Clothing, 6 Dovecote Road Central Point Logistics Park ML1 4GP, U.K.

## Abstract

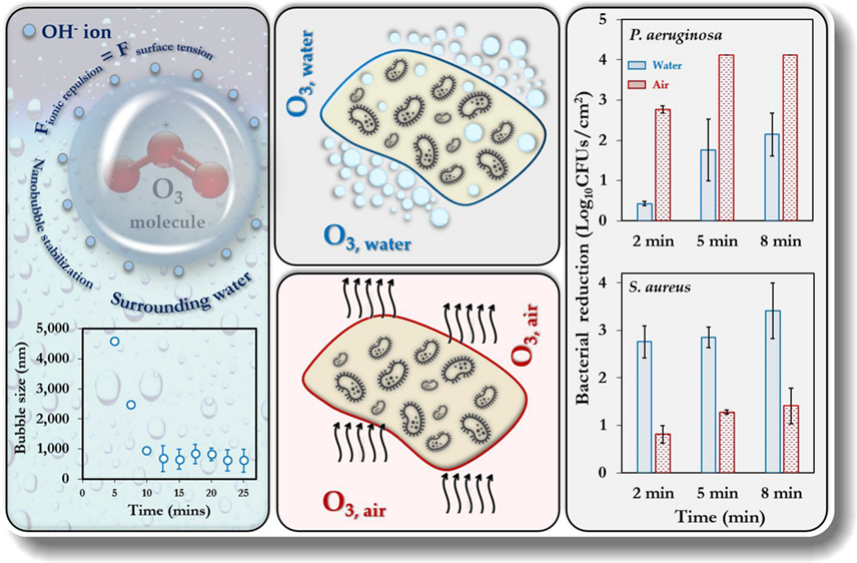

For decades, ozone has been known to have antimicrobial properties when dissolved or generated in water and when utilized in its gaseous form on different substrates. This property (the ability to be used in air and water) makes it versatile and applicable to different industries. Although the medium of ozonation depends on the specific process requirements, some industries have the inherent flexibility of medium selection. Thus, it is important to evaluate the antimicrobial efficacy in both media at similar concentrations, an endeavor hardly reported in the literature. This study provides insights into ozone’s efficacy in air and water using two Gram-negative bacteria (*Escherichia coli* NTCC1290 and *Pseudomonas aeruginosa* NCTC10332), two Gram-positive bacteria (*Staphylococcus aureus* ATCC25923 and *Streptococcus mutans*), and two fungi (*Candida albicans* and *Aspergillus fumigatus*). For gaseous ozonation, we utilized a custom-made ozone chamber (equipped with ultraviolet lamps), whereas an electrolysis oxygen radical generator was applied for ozone generation in water. During gaseous ozonation, the contaminated substrates (fabric swatches inoculated with bacterial and fungal suspensions) were suspended in the chamber, whereas the swatches were immersed in ozonated water for aqueous ozone treatment. The stability of ozone nanobubbles and their resulting impact on the aqueous disinfection efficiency were studied via dynamic light scattering measurements. It was observed that ozone is more effective in air than in water on all tested organisms except *Staphylococcus aureus*. The presented findings allow for the adjustment of the treatment conditions (exposure time and concentration) for optimal decontamination, particularly when a certain medium is preferred for ozonation.

## 1 Introduction

Decontamination is a public health concern as it is key to the prevention of infection transmission, from contaminated materials and surfaces,^1^ particularly in healthcare facilities and in the food industry. It also has important environmental and economic benefits, ensuring the reusability of different materials (via waste and cost reduction). Ozone is an inorganic molecule with powerful antimicrobial properties, attributable to its loosely bonded third oxygen atom (which readily oxidizes other molecules). Its degradative impact on the cell membrane, unsaturated lipids, vital proteins, DNA, and intracellular enzymes of microorganisms has been widely demonstrated.^2−6^

Relative to other popularly applied disinfecting agents (such as steam, ethylene oxide, and ethanol), ozone is one of the few, which can be utilized for decontamination in both gaseous and aqueous forms, and this is one of the reasons for its wide applicability. However, ozone (irrespective of the media in which it is used) is highly unstable and autodecomposes into oxygen with time. While temperature and humidity are key factors affecting ozone’s stability in air,^7^ pH, conductivity, temperature, pressure, and the type of diffuser utilized are key factors, which affect the stability of ozone in water; evidently, more influencing factors (Table 1) are involved with ozone’s aqueous stability and mass transfer efficiency.^8^ Rice et al.^9^ provide some details on different configurations applied to aqueous ozone utilization for commercial laundry applications. In addition, membrane contactors have also been computationally examined as alternatives to conventional gas dispersion methods.^10^

**Table 1:** Comparison of the Practical Considerations Required for Gaseous and Aqueous Ozonation

factor	ozonation in air	ozonation in water
need for drying	keeps substrate dry, eliminating the need for further drying after treatment.	substrate becomes wet and requires drying after treatment, particularly if porous (e.g., textiles).
cleaning	only disinfects or sterilizes the substrate; does not clean it. thus, a separate cleaning step is required before ozonation.	allows for simultaneous cleaning and disinfection. in fact, the use of surfactants has been shown to promote aqueous ozone stability.^29,30^
limitations to ozone generation	depending on the capacity of the generator, higher ozone concentrations (e.g., up to 50 ppm) can be attained rapidly.	mass transfer factors and equilibrium condition (thermodynamic factors) may limit the attainable ozone concentration, relative to ozonation in air, for the same volume and generator capacity.
concentration homogenization	requires efficient gas circulation systems for concentration homogenization.	concentration homogenization strongly depends on efficient gas dispersion in water, often causing high gas usage.
penetration efficiency	better chance of penetration in the gaseous phase for the disinfection of hard-to-reach areas of the substrate.	the efficiency of liquid penetration may be adversely affected for certain substrates (e.g., small-diameter endoscopes).
parameters influencing ozone stability	temperature and humidity are the main influencing factors on the stability of ozone.	the efficiency of the treatment cycle is a function of many variables (pH, conductivity, temperature, pressure, water composition and ozone demand constraints). the generation of nanobubbles enhances ozone stability.
safety	gaseous ozone is detrimental to the lungs when inhaled.	significantly reduced impact on human health when dissolved in water.

Alkaline solutions have been shown to disfavor ozone solubility, as a result of the chain catalytic action of generated OH^–^ ions, whereas better stability is observed in acidic environments. This stability is attributable to the protonation of highly reactive ions, which makes them less active in solution.^11^ Further details of the respective decomposition mechanisms of ozone in air and water can be found in previous studies.^7,12−15^ Moreover, gaseous ozone is usually generated via ultraviolet (UV) radiation or electrical discharge in a closed chamber containing the substrate, to be disinfected. High temperatures and humidity tend to adversely affect gaseous ozone stability,^7^ whereas investigations on the impact of atmospheric pressure on gaseous ozone stability are scarce. However, Kitayama and Kuzumoto^16^ have demonstrated that the efficiency of gaseous ozone generation (via silent discharge) is considerably affected by pressure at low ozone concentrations, although at high ozone concentrations, the effect of pressure is insignificant.

While numerous studies^17−22^ have independently evaluated the impact of ozone’s antimicrobial properties in both air and water using a variety of microorganisms, a comparative assessment of both treatment methods is lacking in the literature. According to the centre for disease control (CDC), more research is required to clarify the effectiveness of ozone mists for the reduction of environmental contamination.^23^ With the recent advent of the COVID-19 pandemic, several contributions have appeared, demonstrating the effectiveness of ozone against the virus.^24−26^ In a bid to provide additional evidence on ozone’s microbial inactivation properties, an investigation of its potency in different states, under similar operating conditions, will be useful and is thus pursued. A recent study by Martinelli et al.^27^ attempts this comparison, but applies dissimilar conditions, particularly in terms of ozone concentration for both treatments. Megahed et al.^28^ also evaluated the microbial killing capacities of gaseous and aqueous ozone on five nonporous materials; however, in this study, we utilize porous substrates (cotton–polyester fabric swatches). The current study provides insights into the optimal deployment of ozone, particularly in industries that have the flexibility of choosing its application medium. Where this flexibility is absent, the presented results allow for the modification of treatment conditions to meet the desired disinfection efficiency.

## 2 Methodology

### 2.1 Substrate Preparation

**Figure 1:**
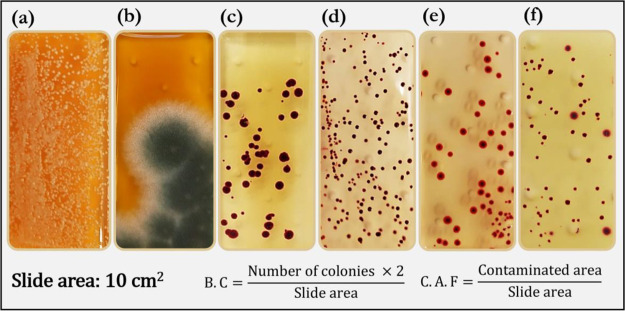
Dipslides used for the enumeration of different organisms applied in this study (a) *C. albicans, CA* (b) *A. fumigatus, AF* (c) *E. coli, EC* (d) *S. aureus, SA* (e) *P. aeruginosa, PA* (f) *S. mutans*, *SA*. BC represents bacterial contamination, whereas CAF represents the contaminated area fraction.

Fabric swatches (35% cotton and 65% polyester) were utilized as the substrates for the evaluation of ozone’s disinfection efficacy in this study. Sterile swatches were inoculated with 100 μL of the bacterial (1 × 10^8^ CFU/mL) and fungal suspensions, which were prepared according to the protocol described in Epelle et al.;^2,31^ however, the substrates were treated in their wet conditions after inoculation. Dipslides (Figure 1) were subsequently applied to the swatches and incubated (at 37 °C for 24–48 h) to evaluate the growth level, pre- and post-ozone treatment. Images of the dipslides were obtained and postprocessed using MATLAB (R2020b) to enumerate the contaminated area fraction for the fungus and the number of formed colonies for the respective bacteria.

### 2.2 Gaseous Ozonation

**Figure 2:**
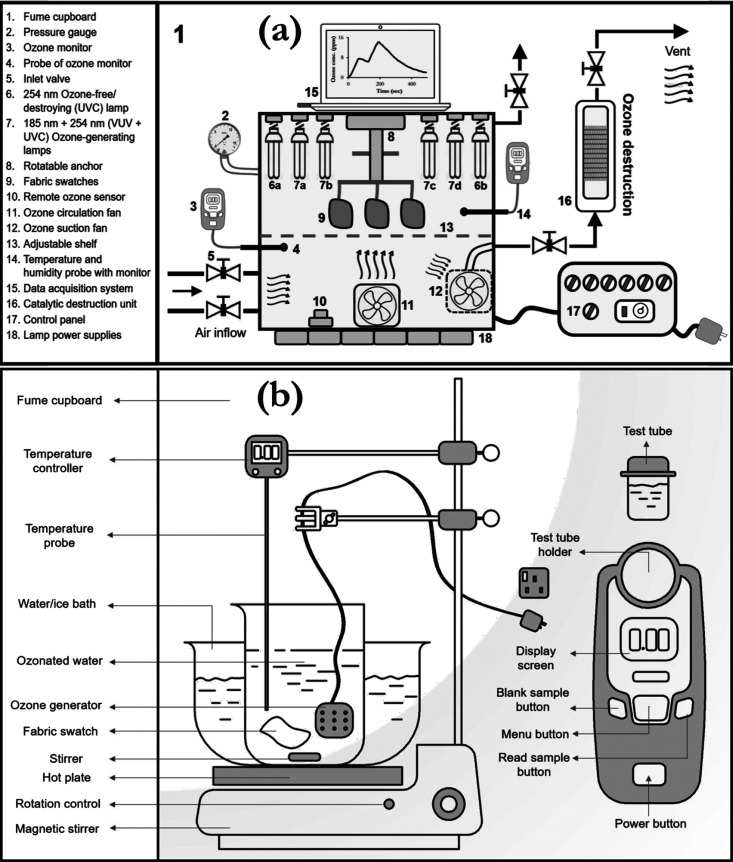
Experimental setup for (a) gaseous ozone disinfection^2^ (Copyright permission from Elsevier. Adapted from Figure 1 in Epelle et al.^2^) and (b) aqueous ozone disinfection.^29^ An accurate comparison was enabled by utilizing the same ozone dosage (ozone concentration × time) and temperatures, for both gaseous and aqueous treatments. The volume of gaseous ozonation chamber is 0.2 m^3^.

A pictorial representation of the chamber used for gaseous ozonation of the contaminated substrates is shown in Figure 2a. The chamber utilizes four low-pressure ozone generating lamps (Jelight Company Inc. USA) for ozone production, which are remotely monitored via an ozone sensor

with a data logging functionality (WinSensors Ltd. China). The chamber is also equipped with two ozone-free lamps for UVC disinfection (although not carried out in this study). The desired concentration for the cycle was maintained by an optimized on/off sequence of the UV lamps in this study. Depending on the number of lamps used, ozone concentrations of up to 30 ppm can be reached in as low as 2.5 min in the chamber (0.2 m^3^). The axial fan (Figure 2a) enables efficient ozone circulation within the chamber, whereas a centrifugal fan, allows for the rapid extraction of the gas into a catalytic destruction unit, upon completing the disinfection cycle. Contaminated fabric swatches can be attached to the shown platform, allowing for good contact between the generated ozone gas and the fabric’s fibers. Further details of the unit are documented in the study of Epelle et al.^2^ The relative humidity in the chamber was 50 ± 2%.

### 2.3 Aqueous Ozonation

Figure 2b illustrates the experimental setup for aqueous ozonation. Ozone was generated via an electrolysis oxygen radical generator (EORGTM – Novus Clean Tech Ltd). Generated ozone was homogenized in the solution via stirring at 100 rpm. The temperature of ozonation was maintained at the same room temperature (18 °C for gaseous ozonation) using a magnetic stirrer equipped with a hot plate and a temperature probe. The Palintest method was employed for ozone concentration measurement, details of which can be found in a previous study.^32^

The contaminated swatches were transferred into 100 mL of ozonated water at the desired concentration and left fully immersed for different durations. After the required contact time was reached, the swatches were transferred onto a sterile surface for the application of the dipslides. The bubble size distribution of 4 ppm ozonated solution, as well as the zeta potential of the generated bubbles, were obtained via dynamic light scattering measurements (Malvern Zetasizer Nano ZEN5600). Equilibration time before the dynamic light scattering (DLS) analysis commenced was set to 120 s. The Smoluchowski approximation for zeta potential calculations was applied, with the Henry function, *F*(κa) = 1.5, for κa > ∼100, where κ is the inverse of the Debye screening length and a is the particle radius. Although nanoparticle tracking analysis has been used in combination with DLS for nanobubble characterization,^33^ we have only utilized DLS measurements in this study.

For both aqueous and gaseous ozonation, the applied ozone doses (concentration × time) were 4, 8, 10, 16, 20, and 32 ppm.min. These corresponded to the application of 2 ppm and 4 ppm ozone concentrations for exposure durations of 2, 5, and 8 min.

### 2.4 Scanning Electron Microscopy Imaging

The preparatory procedure involved transferring 50 μL of the bacterial or fungal suspension onto a sterile fabric swatch, attached to an aluminum stub via double-sided carbon tape. These stubs were placed onto a Petri dish, covered, and incubated for 4 h (at 37 °C) to allow the cells bond onto the fibers of the fabric swatch. This was followed by washing with phosphate buffer saline (PBS – 0.01 M), after which fixation for at least 30 min was performed using a solution of 2.5% glutaraldehyde, 2% paraformaldehyde, and 0.1 M phosphate buffer (pH 7.4). Ethanol dehydration in increasing concentrations (50, 70, 80, 90, 95, and 99% v/v) was subsequently carried out. The samples were further treated with tert-butanol and then freeze-dried (Christ Alpha 1–2 LD plus). Gold sputtering (Emscope SC500) was followed, and thereafter, imaging (Hitachi S-4100) of the samples.

## 3 Results and Discussion

### 3.1 Assessment of Gaseous and Aqueous Ozone Stability by DLS

Before presenting the results of ozone inactivation, it is worth highlighting that the size of the generated ozone bubbles affects ozone stability in the aqueous phase. Compared to conventional bubbling methods, which generate larger bubbles and a consequent short ozone half-life, it was important to ensure that ozone was retained at the desired levels for the required treatment duration, without having to generate ozone again. This stability is in turn a key determinant of the disinfection efficacy. Thus, we briefly discuss the role of nanobubbles in enhancing ozone mass transfer into the aqueous phase, and correspondingly, the stability for sustained antimicrobial action.

**Figure 3:**
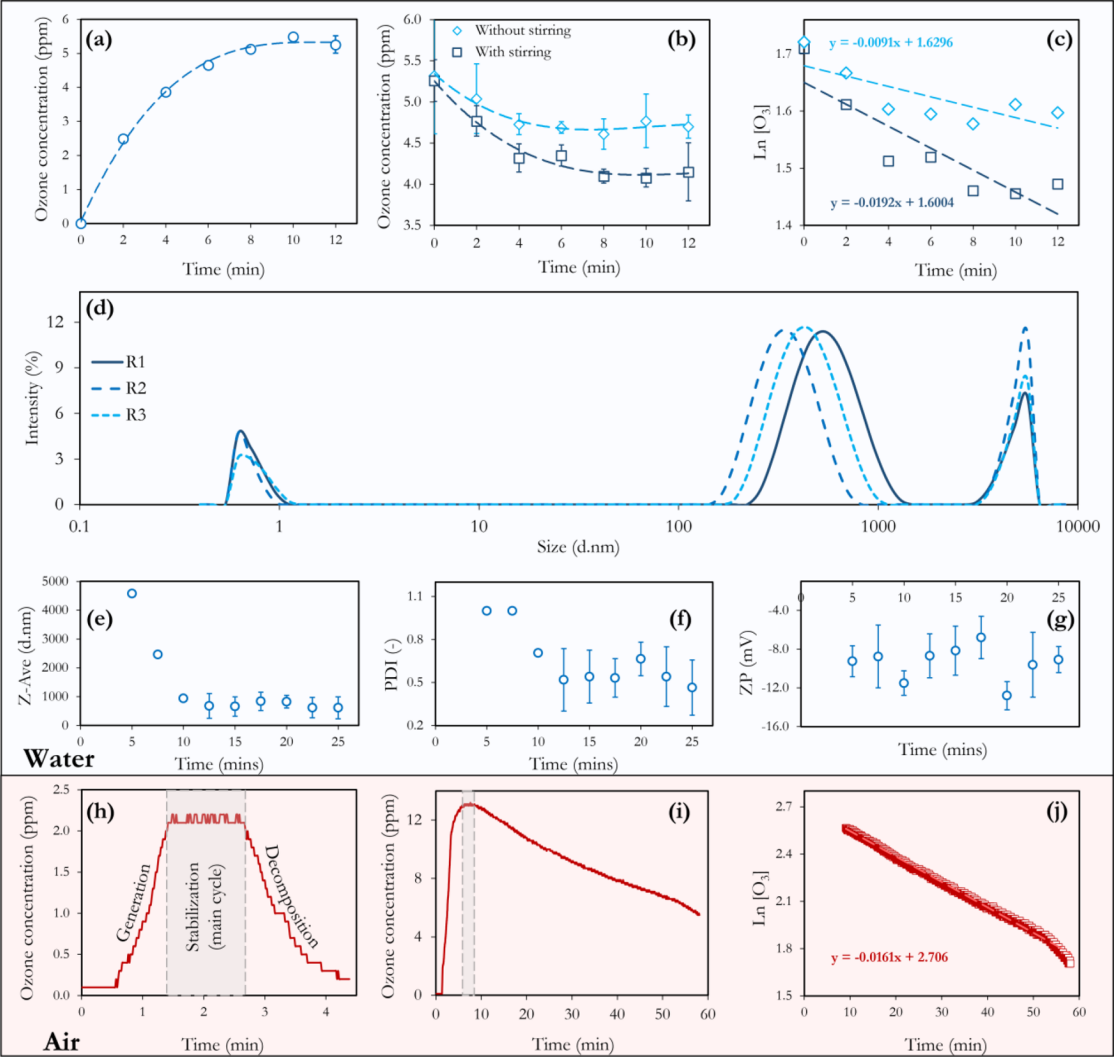
Analysis of aqueous and gaseous ozone stability. Generation (a) and decomposition cycles (b) of ozone in water, with first-order kinetic plots (c). Size distribution of ozone bubbles in solution, at different times after generating 4 ppm ozonated water (d). Three separate runs of the bubble size distribution, which were obtained, ∼10 min after ozonation (e). Variation of ozone bubble polydispersity index (PDI), with time after ozonation (f). Zeta potential variation with time after ozonation (g). Typical gaseous ozone treatment cycle (h), showing the decomposition profile (i) and the first-order ozone decomposition plot (j). Error bars represent the standard deviation of at least three separate measurements.

Ozone generation in 500 mL of water with ionic composition (K = 0.97 mg/L; Ca = 46.95 mg/L; Mg = 17.14 mg/L; Na = 12.45 mg/L; Cl- = 17.23 mg/L; SO_4_^2–^ = 20.66 mg/L; and NO_3_^–^ = 13.95 mg/L) can be observed in Figure 3a. According to the generation profile, the ozone concentration appears to stabilize after 8 min, indicating the attainment of some stabilization. As shown in Figure 3d, the EORG device utilized for aqueous ozonation produces bubbles in the range of 0.8–7000 nm, mostly in the nanobubble, NB (<1 μm) and microbubble, MB (>100 μm) size range based on the classification by Seridou and Kalogerakis.^33^ Bubbles in this size range are governed by Brownian motion, and lower buoyancy forces allow them to stay in the system much longer.

Unlike their macro counterparts, such bubbles have highly enhanced mass transfer properties^34^ and much slower rising velocity with the best results shown by nanobubbles, which can stay in solution for weeks or even months. The prevalence of nanosized bubbles enhances ozone dissolution in the aqueous phase, resulting in the rapid generation rate observed (Figure 3a). Conversely, the effective generation of hydroxyl radicals induced by the collapse of ozone microbubbles enhances aqueous disinfection, particularly because the OH* radical (with a standard redox potential of 2.80 V) is a more powerful oxidant than ozone (2.07 V) itself.^35^

As illustrated in Figure 3b, the aqueous decomposition rates are dependent on the application of stirring; stirring destabilizes the solution, causing ozone to escape, thus yielding the reduced concentration observed. First-order decomposition kinetic plots for aqueous ozone are shown in Figure 3c. The resulting analysis of the slopes indicates aqueous ozone half-lives of 36 and 76 min with and without stirring, respectively. As shown in Figure 3e, the cumulant size (Z-ave) of ozone bubbles tend to reduce over time, eventually forming nanobubbles, which increase ozone gas dissolution. The results show an iteration to an equilibrium diameter (∼700 nm), which happens after 10 min. Hence, the free-radical oxidation of ozone nanobubbles is increased, destroying more microbes in the solution, as will be shown in Section 3.2. This trend of decreasing bubble size can also be observed with the PDI. Five minutes after ozone generation, a wide variation in the size of ozone bubbles can be observed (PDI = 1, Figure 3f); however, this variation generally decreases with time (PDI = 0.46 at 25 min), as shown in Figure 3f, resulting in nanobubbles of more uniform size. This is a further indication of the shrinkage (as induced by surface tension) and potential collapse of microbubbles to form nanobubbles that remain in solution for a longer duration.

The stability of these nanobubbles, as determined by their zeta potential, is shown in Figure 3g. The zeta potential values (average of −10 mV) can be explained by the electron affinity of ozone nanobubbles (negatively charged). According to Li et al.^36^ and Calgaroto et al.,^37^ these bubbles have a strong preference for hydroxide ion (OH^–^) adsorption at the gas–liquid interface; this results in the generation of electrostatic repulsive forces that balance out the surface tension/compressive forces, ultimately preventing the coalescence of the nanobubbles. Furthermore, the nanobubble stability observed (Figure 3b) has also been attributed to this strong electrostatic repulsion between hydroxide ions (which are naturally occurring in the aqueous phase) in the work of Satpute and Earthman.^38^ Because ozone is not an inert gas, there is a potential for its decomposition in the bubble within the timeframes shown in Figure 3b, e. The initial step of ozone decomposition (reaction with the hydroxide ion) has a rate between 40 and 70 M^–1^ s^–1.^^13,14^ Further work is required to ascertain the contribution of ozone decomposition within the bubble to the overall nanobubble size and stability.

The surface charge of the nanobubbles also depends on the pH of the water used. According to Meegoda et al.,^39^ if pH is less than 4, the nanobubbles will obtain a positive charge at the gas-water interface, resulting in the reduction of stability because of high H^+^ ion concentrations; this causes a size increase in the bubbles and consequently their rapid coalescence (H^+^ ions are more hydrated and stay in the aqueous part, while OH^–^ ions are more polarized and attract to the bubble surface). The pH of mineral water utilized in this study (8.07) thus implies better nanobubble stability via increased hydrogen bonding around the bubbles. As a result of the high ionic content of the mineral water utilized for ozone generation, the net electrostatic potential of the slipping plane is “shielded” by additional ions entering the system because of the Debye screening effect as mentioned by Nobbmann.^40^ This is reflected in the observed reduced zeta potential values (Figure 3f) of the nanobubbles relative to those reported by Meegoda et al.^39^

A typical ozone treatment cycle in air is shown in Figure 3h, with the generation phase (which depends on the number of UV lamps), the stabilization phase, and the decomposition phase (which depends on the extraction rate and pressure drop through the catalytic destruct unit). To determine ozone’s stability in air, 14 ppm of ozone was generated in the chamber and left to auto-decompose. Similarly, the first-order decomposition analysis of gaseous ozone was performed (Figure 3j); a half-life of 43 min was observed, which is higher than the stirring scenario in water but lower than the aqueous scenario without stirring (keeping the temperature constant at 18 °C). Thus, the presence of undisturbed nanobubbles may induce better ozone stability in water than in air.

### 3.2 Microbial Inactivation of Gaseous and Aqueous Ozone

Microbial inactivation via ozone can occur directly or indirectly. Direct inactivation involves the oxidizing action of ozone itself, whereas indirect activation involves the reaction of free hydroxyl radicals, which are generated by the decomposition of ozone.^33,41^ While the former is thought to be the dominant mechanism in air, both direct and indirect reactions have significant effects on the resulting disinfection efficiency during aqueous ozonation (particularly at a pH of approximately 7).^42^

**Figure 4:**
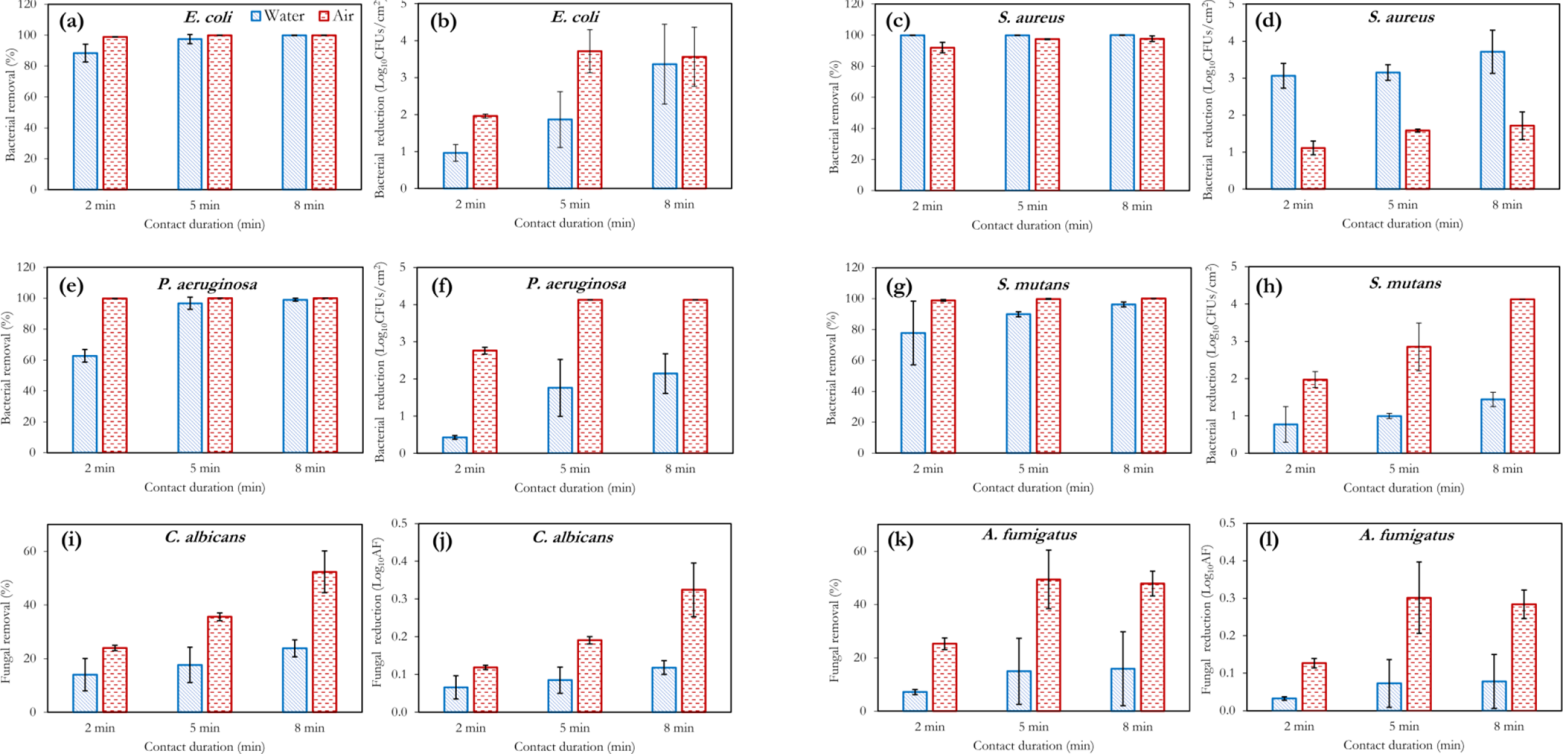
Effect of the contact duration on microbial reduction at **2 ppm ozone concentration** in air and water, for the different microorganisms applied. Microbial log reduction plots are shown beside the percentage reduction plot for each organism to provide better insights into the air-water differences and the variation with respect to time. For gaseous ozonation, RH = 50 ± 2%, whereas *T* = 18 °C for both gaseous and aqueous ozonation.

**Figure 5:**
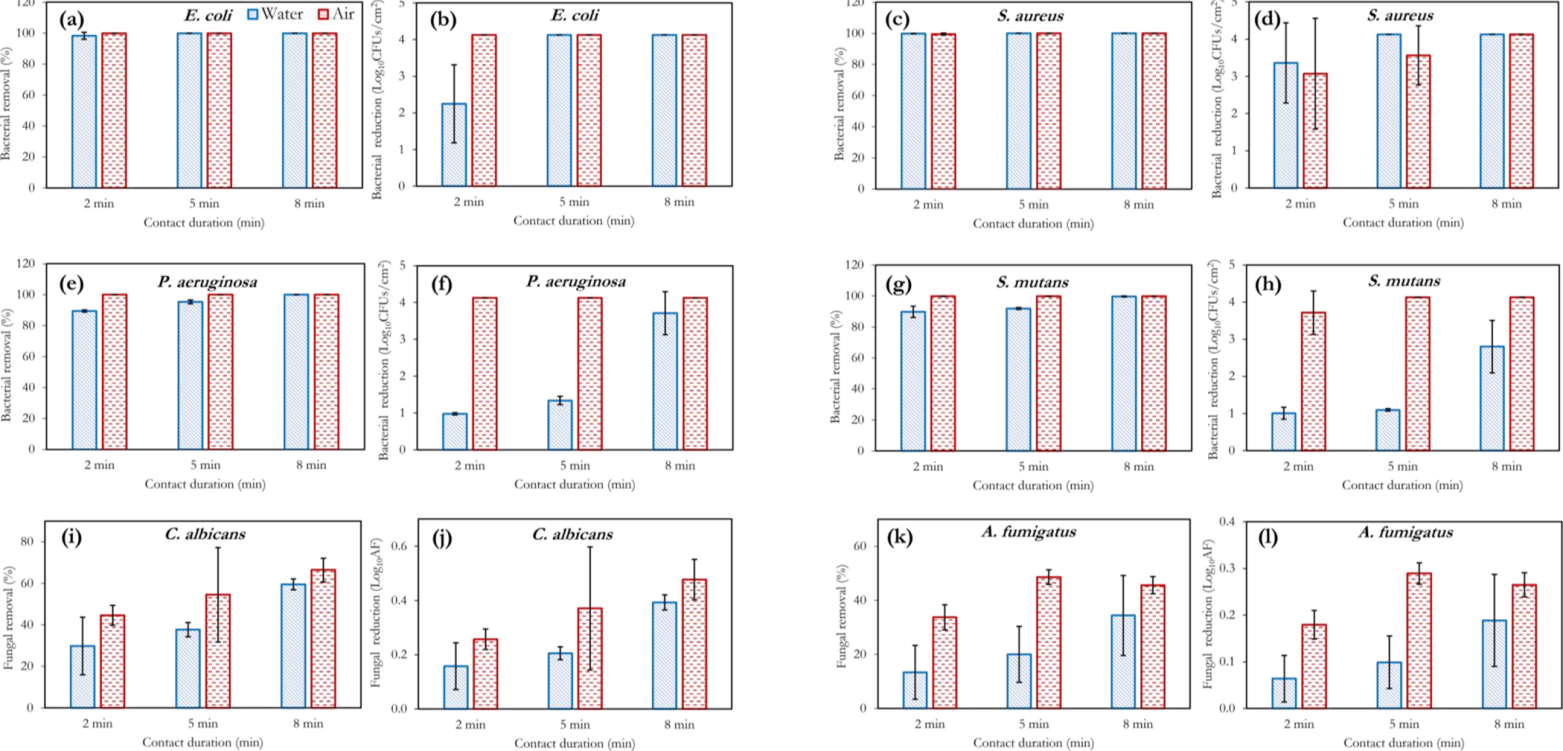
Effect of the contact duration on microbial reduction at **4 ppm ozone concentration** in air and water, for the different microorganisms applied. Microbial log reduction plots are shown beside the percentage reduction plot for each organism to provide better insights into the air-water differences and the variation with respect to time. For gaseous ozonation, RH = 50 ± 2%, whereas *T* = 18 °C for both gaseous and aqueous ozonation.

Figures 4 and 5 illustrate the microbial inactivation potency of ozone on 6 different organisms at concentrations of 2 and 4 ppm, respectively. With aqueous ozonation, the general observable trend is the improvement in the inactivation efficiency with contact duration. As previously highlighted, this inactivation is attributable to the presence of ozone micro- and nanobubbles, which have a longer residence time in aqueous solutions compared to macrobubbles that tend to rapidly rise to the air-water interface and collapse. This longer residence time may be ascribed to their increased gas–liquid interfacial area, reduced buoyancy, and resistance to coalescence.^43^ This translates to an increased oxidation ability and in turn the reasonable disinfection efficiency observed herein. For all tested organisms, *S. aureus* had the least resistance to aqueous ozone treatment with >3 Log_10_ reductions attained at 2 ppm in 2 min (Figure 4d), whereas *A. fumigatus* proved very difficult to inactivate at both ozone concentrations and exposure/treatment durations; only 50% reduction (<0.3 Log_10_ reductions) was attained at 4 ppm ozone exposure for 8 min (Figure 4l). Furthermore, *S. mutans* was the most resistive bacteria to aqueous ozone (Figure 4h); complete removal could only be attained at 4 ppm exposure for 8 min (Figure 5h). It should also be pointed out that although free radical generation complements the inactivation process, it is also a prolific ozone decomposition site, as explained by the reaction mechanisms of Tomiyasu et al.^13^ and Staehelin et al.^14^ Given the higher oxidation potential of OH* radicals compared to ozone, it then becomes necessary to ascertain the OH* production rate in solution, relative to the O_3_ removal rate at different pH levels, particularly because other species with reduced microbial effects are also formed during ozone’s decomposition.

Ozone’s action in air is also shown in Figures 4 and 5, with a similar inactivation trend to that of aqueous ozone observed. *P. aeruginosa* showed the least resistance to gaseous ozone in this study; 2 ppm exposure for 5 min was sufficient to achieve complete inactivation of this bacteria (Figure 4e). As with the aqueous scenario, *A. fumigatus* proved the most difficult for gaseous ozone inactivation (Figures 4l and 5l). In fact, the increase in the ozone concentration from 2 to 4 ppm made no significant difference/improvements to the inactivation of this fungus. However, Epelle et al.^2^ have demonstrated that up to 20 ppm is required to achieve complete inactivation of this fungus for the same inoculum volume applied herein. A general improvement in the inactivation efficiency is observable across all other organisms (*EC*, *SA*, *PA*, *SM*, and *CA*) when the ozone concentration in the aqueous and gaseous phases is increased from 2 to 4 ppm. However, doubling the concentration does not in turn create a two-fold increase in the disinfection efficiency, as there exists a critical concentration and exposure time required for the complete inactivation of each organism. For a substrate contaminated with all 6 organisms, the results have shown that a higher ozone concentration (>4 ppm) or a longer exposure duration is required for sterilization. Although the direct inactivation scenario is thought to be the main route for gaseous ozone inactivation in this study, the generation of OH* radicals cannot be ruled out, particularly because of the application of UV radiation for ozone generation using air as the feed gas (which naturally contains water vapor). However, the OH* generation rate is thought to be significantly lower than that in water.

A major observable trend in Figures 4 and 5 is the superior microbial inactivation of gaseous ozone generation to aqueous ozonation for all organisms utilized except *S. aureus*. A similar observation was also made in the work of Martinelli et al.^27^ Besides the action of ozone, the drying effect produced by the axial fans (utilized continuous ozone gas circulation) is a possible contributor to the enhanced death rate of the organisms (they are more likely to thrive under wet than under dry conditions). However, relative to other organisms, *S. aureus* and its Methicillin-resistant strains have been reported to possess a marked resistance to drying^44,45^ this may be a possible reason for the opposite behavior (aqueous better than gaseous ozonation) observed for this bacteria. In addition, the gas–liquid mass transfer limitations of aqueous ozonation are nonexistent in air; thus, the penetration of gaseous ozone into the fibers of the swatches harboring the bacteria is enhanced compared to a scenario where the dissolved gas (via micro/nano bubble entrapments) faces a liquid (water) and solid (fabric swatch) barrier. Fichet et al.^46^ demonstrated the superior inactivation characteristics of gaseous hydrogen peroxide (>4 Log_10_ reduction), against prions relative to liquid peroxide treatment (≤1 Log_10_ reduction). They attributed this observation to the increased reactivity of the gaseous peroxide and the greater penetration into target molecules. It was argued that liquid peroxide treatment induces the formation of multimers and other solid constituents, which protect the target pathogens and inhibit ozone penetration. Although the fabric and water utilized were sterile, this does not exclude the possibility of other microscale constituents in solution, which eventually may have acted as shields in the solution against the penetration of dissolved ozone.

It is important to mention that Megahed et al.^28^ reported a different observation, the superiority of aqueous ozone treatment over gaseous treatment of nonporous substrates contaminated with cattle manure. Similar observations are also reported by Tizaoui et al.^47^ against the SARS-CoV-2 virus. This indicates that the nature of the substrate (porous or non-porous) and the type of microorganism utilized affect the performance of gaseous and aqueous ozonation. We hypothesize that the porosity and wetness of the swatches utilized in this study yield a more balanced contribution from both direct and indirect oxidation routes. This favors the inactivation efficiency of gaseous ozone, compared to aqueous ozone in which indirect oxidation via OH* radicals is the likely dominant inactivation mechanism. Furthermore, the shielding effect of bacterial cells (in clumps) dried onto nonporous surfaces and biofilms are more readily weakened by aqueous conditions for ozone action, compared to dry gaseous ozonation. This is a likely reason for the observations reported in these studies.

**Figure 6:**
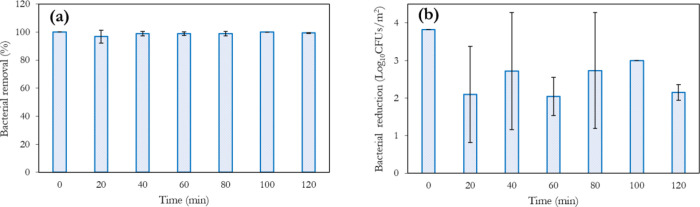
Retainment of the biocidal properties of ozonated water against *E.coli*. The initial concentration of aqueous ozone utilized is 4 ppm.

It was also of interest to examine the biocidal retention capability of ozonated water over time. As can be observed in Figure 6, >2 log reduction in *E.coli* is observed up to 2 h after the generation of 4 ppm ozonated water. This finding of prolonged biocidal activity of ozonated water against *E.coli* can be attributed to the nanobubble stability in solution. A similar analysis by Seki et al.^48^ has shown tremendous stability (up to 1 week) with antimicrobial properties retained. This demonstrates the utilization of aqueous ozone (at controlled concentrations) as a potential hand or surface disinfectant, as is currently done with ethanol solutions, particularly relevant in the face of the COVID-19 pandemic. A comparative assessment of the antimicrobial benefits of both disinfectants, as well as the health and environmental risks, will be worthy of future investigation.

### 3.3 Analysis of SEM Images

**Figure 7:**
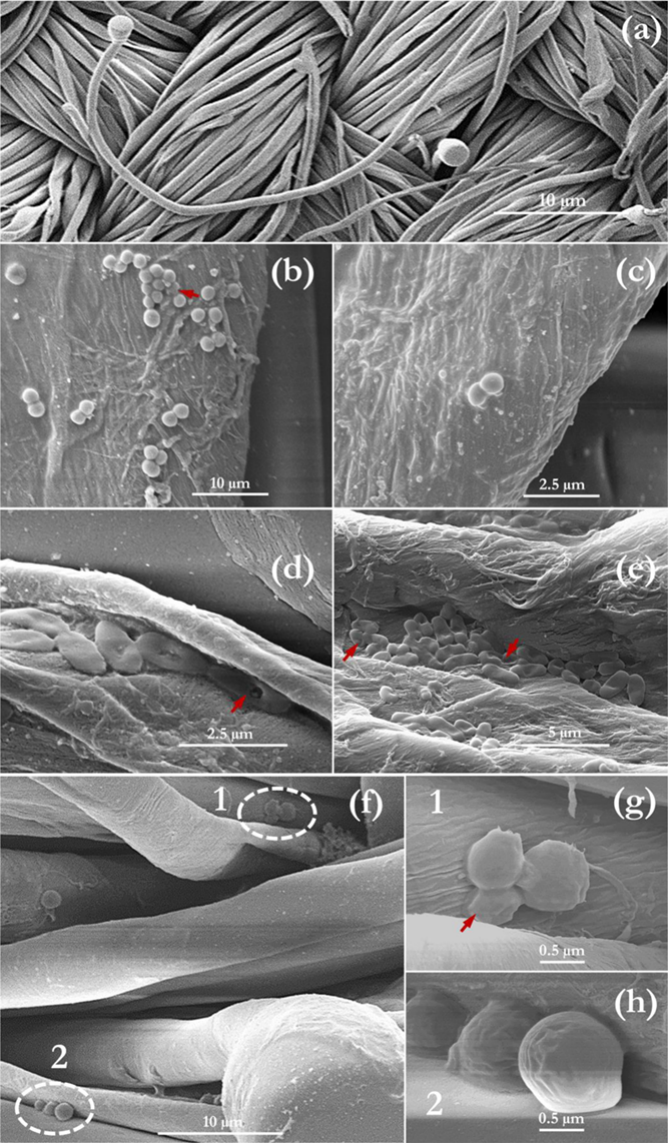
SEM of the fabric swatch used in this study (the substrate for disinfection (a); ozone treated fabric inoculated with *S. aureus* (b); *S. mutans* (c); *P. aeruginosa* (d) *E. coli* (e); and *C. albicans* (f–h)). Red arrows indicate regions of cell damage by the action of gaseous ozone (10 ppm for 10 mins). In f, regions 1 and 2 are magnified to give g and h.

Figure 7 illustrates the interaction of some tested microbes with the fabric swatch used for disinfection herein. Compared to a flat polished surface (in which the cells can be readily located), the use of a fabric swatch increases the difficulty of finding bacterial cells, as a result of the prominent appearance of the fibers. Furthermore, the cells of the different organisms showcased different positional behaviors relative to the fabric swatch. It can be observed that spherically shaped cells, such as those of *S. aureus*, *S. mutans*, and *C. albicans*, tend to be positioned on top of the fibers, whereas ellipsoidal cells (*E. coli*, *P. aeruginosa*) were mostly aligned with the general fiber orientation and occasionally situated in between two fibers or within the opening/crack of a single fiber. This may have also contributed to the superior performance of gaseous ozonation on these bacteria, as the penetration into all areas is more probable in air than with water. It can also be observed that the action of ozone on the cells was mainly a deformation of their structure, as shown by the red arrows in Figure 7. This deformation (mainly flattening and roughening of the cell membrane) was mainly observed in the ellipsoidal/Gram-negative bacterial cells (*E. coli* and *P. aeruginosa*) compared to the spherical Gram-positive bacterial cells (*S. aureus* and *S. mutans*).

Inevitably, this can be attributed to the thin peptidoglycan cell wall and the lipopolysaccharide outer membrane of the Gram-negative bacteria, compared to the far thicker peptidoglycan layers of the Gram-positive bacteria. A previous study by the authors^2^ illustrates the oxidation of the cell wall of *E. coli*, with severe leakage of the cell constituents. This is often followed by the damage of the nucleic acids (purines and pyrimidines) and the breakage of the carbon-nitrogen bonds, leading to further cell lysis.^49^ Based on the obtained SEM results, we conclude that cell wall rupture is not a compulsory step during gaseous ozone inactivation (particularly for gram-positive bacteria). No viable cells were recovered from the swatch after ozone treatment prior to SEM imaging. Thus, RNA and DNA breakdown, protein coagulation, and the degradation of intracellular enzymes^3,50−52^ after the diffusion of ozone into the cell are believed to be other inactivation mechanisms that may have led to microbial inactivation. This absence of structural damage via dry gaseous ozone has also been demonstrated in the work of Mahfoudh et al.^53^ In contrast, humidified gaseous ozone was found to induce spore swelling, facilitating the diffusion of oxidative species into the cell for inactivation. This was also demonstrated in the SEM micrographs of dos Santos et al.,^8^ where ozonated water was applied.

## 4 Conclusions

In this study, the microbial inactivation efficiency of gaseous and aqueous ozonation has been evaluated under the similar conditions (exposure duration, ozone concentration, and temperature), using two Gram-negative bacteria (*E. coli* and *P. aeruginosa*), two Gram-positive bacteria (*S. aureus* and *S. mutans*), and two fungi (*C. albicans* and *A. fumigatus*). The obtained results have shown superior performance of gaseous ozonation over the application of ozonated water for all organisms tested except *S. aureus*. We attribute this performance to the increased ozone penetration attainable in the gaseous phase, relative to the aqueous scenario that is plagued by gas–liquid mass-transfer constraints.

*P. aeruginosa* showed the lowest resistance to gaseous ozonation, whereas *S. aureus* had the lowest resistance in ozonated water. SEM observations of the cell morphology suggest that the ellipsoidal cells (mainly of the Gram-negative bacteria) are prone to cell wall degradation by ozone, compared to the spherically shaped cells of the Gram-positive bacteria. Furthermore, the antimicrobial properties of ozonated water at 4 ppm are still retained after 2 h. This may be attributed to the presence of ozone nanobubbles in the aqueous solution, aiding ozone dissolution and prolonging its antimicrobial properties. Besides the size-distribution analysis illustrating the marked presence of ozone nanobubbles, the obtained negative zeta potential values further substantiated their presence in solution.

However, it is important to point out that the ozone generation methods (in air and water) utilized in this study may generate other reactive species; thus, it is difficult to completely exclude their effects on the inactivation efficiencies reported. Although the use of a pure oxygen feed (instead of air) as the precursor for gaseous ozone generation is likely to mitigate this problem, that of water is more complex. As such, we recommend that further investigations take this into consideration and expand the parameter space to include more ozone doses, pH values, RH, temperatures, and different materials/substrates; the impact of other ozone generation methods (corona discharge and bubble diffusion techniques) is also worth investigating. Nonetheless, the findings presented herein allow for the optimal industrial deployment of gaseous and aqueous ozonation for effective disinfection. In addition to the disinfection efficiency, other factors (as mentioned in Table 1) come into play when deciding on the ozonation medium and should be considered. It will be of interest particularly to the textile industry to investigate the influence of gaseous and aqueous ozone treatment on the mechanical integrity of textile fibers. In the long run, this affects their longevity, reusability, and the reduction of clothing waste. This will facilitate the optimal design of ozone-contacting equipment for large-scale disinfection purposes.

## Symbols & Acronyms

AF: *Aspergillus fumigatus*
BC: Bacterial contamination
CAF: Contaminated area fraction
CFU: Colony forming unit
CA: *Candida albicans*
EC: *Escherichia coli*
PA: *Pseudomonas aeruginosa*
PDI: Polydispersity index
SA: *Staphylococcus aureus*
SM: *Streptococcus mutans*
Z-Ave: Average bubble diameter (nm)
ZP: Zeta potential (mV)
